# Progression-specific genes identified in microdissected formalin-fixed and paraffin-embedded tissue containing matched ductal carcinoma in situ and invasive ductal breast cancers

**DOI:** 10.1186/s12920-018-0403-5

**Published:** 2018-09-20

**Authors:** Silke Schultz, Harald Bartsch, Karl Sotlar, Karina Petat-Dutter, Michael Bonin, Steffen Kahlert, Nadia Harbeck, Ulrich Vogel, Harald Seeger, Tanja Fehm, Hans J. Neubauer

**Affiliations:** 1 Department of Obstetrics and Gynaecology, Life-Science-Center, Heinrich-Heine University, Merowingerplatz 1A, 40225 Duesseldorf, Germany; 2 Institute of Pathology, Department of Pathology, Ludwig Maximilians University, Thalkirchner Straße 36, 80337 Munich, Germany; 3 Microarray Facility, Department of Medical Genetics, Eberhard Karls University, Tuebingen, Germany; 4 IMGM Laboratories GmbH, Bunsenstr. 7a, 82152 Martinsried, Germany; 5 Department of Obstetrics and Gynaecology, Ludwig Maximilians University, Marchioninistr. 15, 81377 Munich, Germany; 6 Institute of Pathology, Eberhard Karls University, Tuebingen, Germany; 7 Department of Obstetrics and Gynaecology, Eberhard Karls University, Liebermeisterstr. 8, 72076 Tuebingen, Germany; 8 Department of Obstetrics and Gynaecology, Eberhard Karls University, Calwerstr. 7, 72076 Tuebingen, Germany; 9 Department of Obstetrics and Gynaecology, Heinrich-Heine University, Duesseldorf, Moorenstr. 5, 40225 Duesseldorf, Germany

**Keywords:** Breast cancer, Pure DCIS, Gene expression, Laser microdissection, Matched pairs, FFPE samples

## Abstract

**Background:**

The transition from ductal carcinoma in situ (DCIS) to invasive breast carcinoma (IBC) is an important step during breast carcinogenesis. Understanding its molecular changes may help to identify high-risk DCIS that progress to IBC. Here, we describe a transcriptomic profiling analysis of matched formalin-fixed and paraffin-embedded (FFPE) DCIS and IBC components of individual breast tumours, containing both tumour compartments. The study was performed to validate progression-associated transcripts detected in an earlier gene profiling project using fresh frozen breast cancer tissue. In addition, FFPE tissues from patients with pure DCIS (pDCIS) were analysed to identify candidate transcripts characterizing DCIS with a high or low risk of progressing to IBC.

**Methods:**

Fifteen laser microdissected pairs of DCIS and IBC were profiled by Illumina DASL technology and used for expression validation by qPCR. Differential expression was independently validated using further 25 laser microdissected DCIS/IBC sample pairs. Additionally, laser microdissected epithelial cells from 31 pDCIS were investigated for expression of candidate transcripts using qPCR.

**Results:**

Multiple statistical calculation methods revealed 1784 mRNAs which are differentially expressed between DCIS and IBC (*P* < 0.05), of which 124 have also been identified in the gene profiling project using fresh frozen breast cancer tissue. Nine mRNAs that had been selected from the gene list obtained using fresh frozen tissues by applying pathway and network analysis (MMP11, GREM1, PLEKHC1, SULF1, THBS2, CSPG2, COL10A1, COL11A1, KRT14) were investigated in tissues from the same 15 microdissected specimens and the 25 independent tissue samples by qPCR. All selected transcripts were also detected in tumour cells from pDCIS. Expression of MMP11 and COL10A1 increased significantly from pDCIS to DCIS of DCIS/IBC mixed tumours.

**Conclusion:**

We confirm differential expression of progression-associated transcripts in FFPE breast cancer samples which might mediate the transition from DCIS to IBC. MMP11 and COL10A1 may characterize pure DCIS with a high risk developing IDC.

**Electronic supplementary material:**

The online version of this article (10.1186/s12920-018-0403-5) contains supplementary material, which is available to authorized users.

## Background

Breast cancer is the most common malignancy among women in western countries. Its incidence has been increasing since the 1940’s [1]. The prevailing concept of a multi-step model of mammary carcinogenesis is a sequence of pathologically defined stages beginning with atypical ductal hyperplasia (ADH) which is progressing to the preinvasive ductal carcinoma in situ (DCIS), a non-obligate precursor of the final stage, invasive breast carcinoma (IBC) [2, 3].

DCIS accounts for approximately one-third of all newly diagnosed breast cancer cases. It has been estimated that about 50% of untreated DCIS lesions will progress to IBC with wide variations in latency [4, 5]. Furthermore, half of the local recurrences that develop after initial surgical treatment of DCIS are invasive cancers [6]. Women diagnosed with DCIS have an estimated 4 to 12 times higher relative risk of developing invasive breast cancer [7], whereas on the other hand, some untreated DCIS will change very little within 5–20 years [8]. Given that only a minority (15–30%) of women diagnosed with pure DCIS (pDCIS) develop subsequent breast tumour within the first decade after treatment with lumpectomy alone, and that approximately 70% of women with pDCIS are treated with lumpectomy in conjunction with radiation and antihormonal treatment, it is likely that many women with pDCIS are being overtreated [9, 10]. Thus, there is a clinical need to identify (a) prognostic biomarker(s) that accurately predict the clinical behaviour of DCIS and that support the clinician to select the most appropriate treatment regimen. Patients expected to develop indolent disease could then be treated with lumpectomy alone, whereas those expected to develop invasive disease could receive a more aggressive treatment.

The traditional grading of DCIS is based on morphologic features [11]. However, considerable heterogeneity limits classical histopathology’s ability to accurately predict the risk of progression from DCIS to IBC so that it has only limited clinical value [8, 12, 13]. Invasive carcinomas have been reported to develop from DCIS of all nuclear grades [4], which supports the idea of an invasive phenotype beyond histopathological and molecular intrinsic subtypes. Understanding the molecular biology of DCIS and its transition to IBC may provide insight into tumour initiation and progression and may enable the identification of biologically and clinically significant progression-associated genes. We and others have published transcripts that are differentially expressed between matched DCIS and IBC and which may be able to predict the probability of DCIS progressing to IBC [14–18].

In the current study we used formalin-fixed, paraffin-embedded (FFPE) tissue samples from 15 patients with both DCIS and IBC (FFPE investigation). Tumour cells were – as in the first study - laser microdissected (laser capture microdissection, LCM) and extracted mRNA was expression profiled using the Whole Genome cDNA-mediated Annealing, Selection, Extension, and Ligation (DASL)-Assay - a technique developed to quantify degraded RNA samples [19]. By combining results from this FFPE investigation with data from the earlier fresh frozen investigation we identified 124 overlapping transcripts. Differential expression of nine transcripts selected based on data obtained from the initial fresh frozen experiment could be independently confirmed in an additional FFPE tissue validation set consisting of 25 independent laser microdissected DCIS/IBC sample pairs. By extending the expression validation to 31 microdissected pDCIS, two potential transcripts were identified whose expression is continuously increasing from pDCIS to DCIS of DCIS/IBC mixed tumours and further to IDC and might characterize pDCIS associated with a high-risk of progression to invasive disease.

## Methods

### Formalin-fixed and paraffin-embedded tumour tissue

For expression analysis paraffin blocks from 15 surgically excised primary breast cancer specimens containing both, DCIS and IBC areas, were obtained from the Department of Pathology of the University Hospital, Tuebingen, Germany. All surgical procedures had been carried out in the Department of Obstetrics and Gynaecology of the University Hospital, Tuebingen. Tissue samples were fixed in buffered formalin after surgical excision and embedded in paraffin according to standard procedures. All tissue samples were obtained with the patients’ informed consents (ethical consent of the Medical Faculty Tuebingen: AZ.266/98). Cryopreserved tissue from five tumours had been studied in our earlier fresh frozen investigation [18].

For validation analysis FFPE tissues from further 25 patients with DCIS/IBC tumours and 31 patients with pDCIS were selected from the archives of the Department of Pathology, Ludwig Maximilians University, Munich. In keeping with local Ethics Committee guidelines, tissue blocks were anonymized.

The Van Nuys grading system for DCIS, in which DCIS is defined by nuclear grade [20], was applied. The predominant growth patterns were solid, cribriform, and papillary. The lesions were of nuclear grades 2 and 3. Patients with pDCIS cases were free of associated invasive components and did not have evidence of recurrence or progression to invasive disease within the 5 years prior to sample processing. It is acknowledged that DCIS may recur after long periods of time, and we cannot rule out that recurrences may be found in the pDCIS group with longer follow up, although the overall long-term recurrence rate for properly excised low grade DCIS is less than 5%. The majority of patients underwent lumpectomy (+/− re-excision), and most of these patients received postoperative radiotherapy.

### Prosigna™ breast Cancer prognostic gene signature assay

The Prosigna™ Assay measures the expression of 58 genes including reference genes to classify the tumour sample into one of the four intrinsic subtypes and to give information about the risk of recurrence (ROR). For determination of the ROR category of each tumour sample the ROR score is separated into three risk groups: low (ROR score 0–40), intermediate (ROR score 40–60) and high (ROR score 60–100, [21]. The assay was used according to the manufacturer’s conditions. Briefly, 250 ng total RNA of FFPE samples was captured by barcoded reporter probes and hybridized overnight (65 °C) to the surface of the Prosigna™ test cartridge. After immobilization of the RNA-probe-complex the expression of the genes was determined by counting the barcodes using the nCounter® Analysis System (NanoString Technologies®).

### Laser capture microdissection (LCM)

Laser capture microdissection of FFPE tissue was performed in order to enrich for tumour cells for subsequent molecular analysis. In brief, serial FFPE sections were cut at 5–8 μm and mounted on special 2-μm-thick membrane slides (MMI AG, Glattbrugg, Switzerland), dried for 10 min at 50 °C and stored at − 20 °C until further deparaffinization and staining. To preserve RNA integrity, these two steps were kept as short as possible. To protect the RNA from contamination and nuclease degradation, the whole system (including microtome and water bath) was cleaned with RNaseZap (Ambion, Austin, USA) or heated to 180 °C for 4 h, and only RNase-free reagents were used. To avoid cross contamination, the microtome blade, DEPC water and chemicals were renewed before processing each new FFPE block. Deparaffinization was achieved by immersion of the slide-mounted section in xylene (2 × 10 min) and pure ethanol (2 × 2 min). The sections were then rehydrated for 1 min and stained with hematoxylin QS counterstain (Linaris GmbH, Dossenheim, Germany) for 10 s. Finally, the specimen were briefly washed in RNase-free water, dehydrated in pure ethanol for 3 min and air-dried for 30 min.

**Fig. 1:**
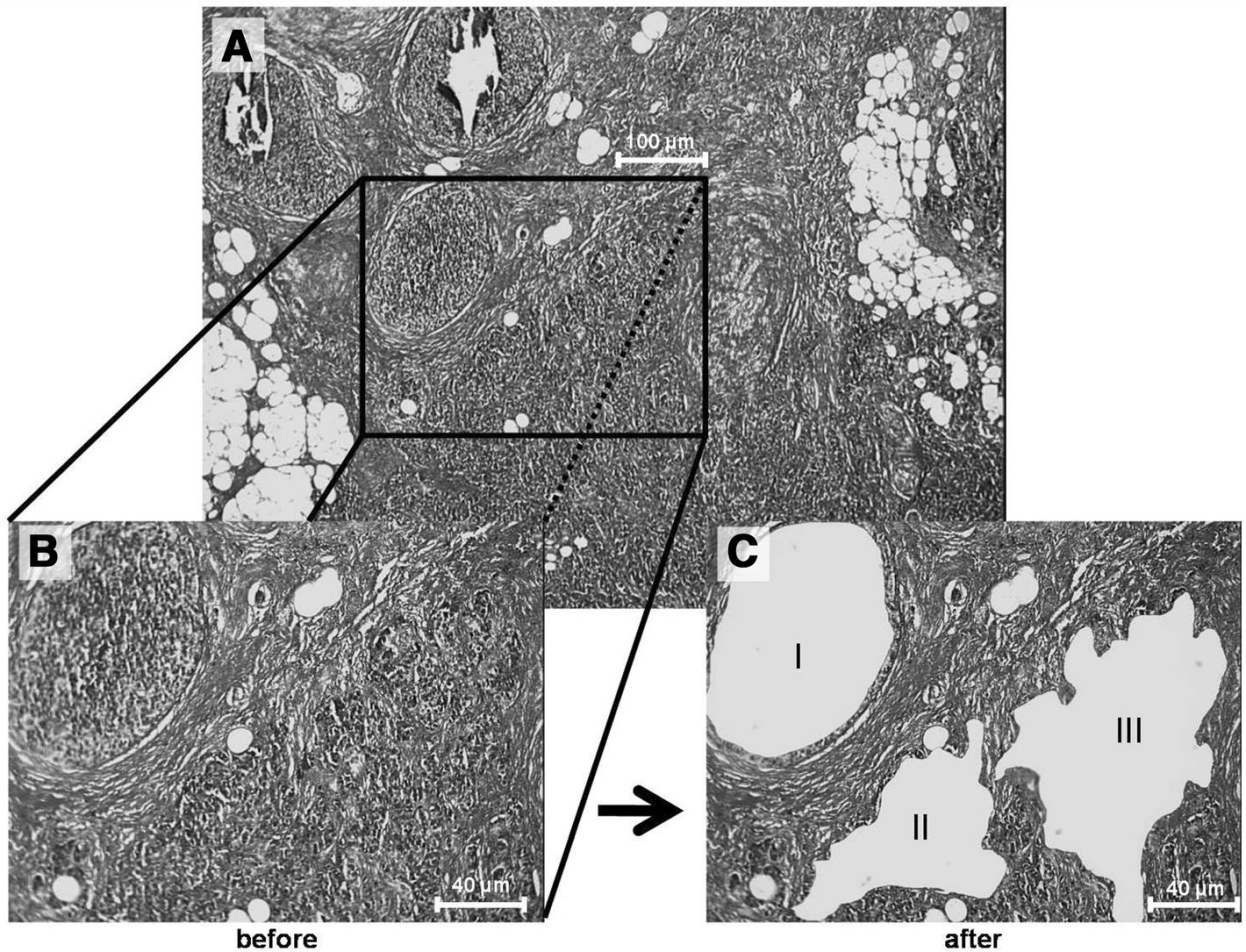
Laser capture microdissection. Depicted are images of stained tissue section before (**a**, **b**) and after (**c**) LCM in a representative breast cancer. I = DCIS area after LCM, II & III = IBC areas after LCM. Magnification A) 4×; B and C) 10×. Size Bar A) 100 μm; B and C) 40 μm

LCM was performed with the MMI μCut system (MMI AG, Glattbrugg, Switzerland). A representative section before and after the LCM procedures is shown in Fig. 1. Depending on the extent of intervening stroma, areas ranging from 1 to 20 μm^2^ (approx. 10,000 to 200,000 cells) were dissected, placed into 30 μl lysis buffer (High pure miRNA isolation kit, Roche, Mannheim, Germany) and stored at − 80 °C until further use.

### RNA extraction

Total RNA was isolated with the High pure miRNA isolation kit (Roche, Mannheim, Germany) according to the one-column protocol. In brief, harvested cells were lysed in lysis buffer and digested with proteinase K (1 mg/ml) overnight (55 °C). Then binding buffer and binding enhancer were added. The mixture was transferred to a High pure filter tube and centri-fuged (30 s, 13,000 xg). After washing, total RNA was eluted and quantified with NanoDrop Spectrophotometer ND-1000 (NanoDrop Tech., Wilmington, USA). Approximately 20–100 ng of total RNA was retrieved by RNA extraction.

### Whole genome DASL assay

About 200 ng mRNA was hybridized to the Whole-Genome Gene Expression DASL-Array (Illumina Inc., San Diego, USA) according to the manufacturer’s instructions. This system uses priming with random hexamers instead of oligo(dT) priming to achieve higher detection rates with RNA extracted from FFPE tissue [22]. After cDNA synthesis and biotinylation, the resultant cDNA was connected with assay-specific oligonucleotides to bind to paramagnetic beads. Following PCR using fluorescence-labelled primers, the amplification products were hybridized to the Whole-Genome Expression BeadChip and scanned by BeadArray Reader (Illumina Inc., San Diego, USA). The GEO database accession number for this dataset is GSE72205. Eleven of the 31 selected pDCIS have also been investigated by DASL-Array and results are included in the GSE data set.

### Statistical analysis of microarray data

Statistical analysis of the results of the FF investigation was performed as described previously [18]. To analyse the DASL array data the intensities of replicate beads and quality control were averaged with Genome Studio V2009.1 software (Illumina, San Diego, CA). No background correction or normalization was performed at this stage [23]. Summarized intensities together with standard errors, number of beads per bead type and detection *p*-values were exported. All subsequent data analysis steps were performed on the software platform R 2.12.0 and Bioconductor 2.6.1 [24] with the packages ‘beadarray’ [25, 26], ‘limma’ [27], ‘RankProd’ [28] and ‘GOstats’ [29]. Initially, the expression data from all chips were normalized with VSN [30]. Non-informative probes were removed from the data set. The differences in mRNA expression levels between DCIS and IBC were analysed by three different approaches. First, linear modelling was used as a parametric approach. The factors *tumour stage* (DCIS/IBC) and *patient* were used to design a linear model capturing the influence of the different factors on gene expression levels. The coefficients describing the expression profiles of the remaining probes were calculated and the standard errors were determined using an empirical Bayesian approach. From the t-statistic the resulting *p*-values were established and corrected for multiple testing by the Benjamini-Hochberg procedure [31]. Secondly, two non-parametric tests (Wilcoxon Signed Rank and Rank Products, [28]) were applied to identify differentially expressed genes. The expression values of the DCIS samples were subtracted from the expression values of the IBC samples and the rank products were calculated as a one class case with 1000 permutations.

### RNA amplification and quantitative real-time PCR (qPCR)

In order to provide enough mRNA for further investigation of the expression of several candidate genes from the limited amount of mRNA obtained from LCM tissue, total RNA (80–100 ng) was pre-amplified using the WT-Ovation™ FFPE RNA Amplification System V2 according to the manufacturer’s instructions (Nugen™ Technologies, San Carlos, USA). The reactions comprise first strand cDNA synthesis, second strand cDNA synthesis and Ribo-SPIA™ amplification cycles. PCR primers were designed using the Primer3 software (http://frodo.wi.mit.edu/primer3) and ordered at biomers.net (Ulm, Germany). Primer sequences are listed in Table 1. QPCR was performed with the LightCycler® 480 System using the LightCycler® 480 SYBR Green Master I (Roche, Mannheim, Germany) and the following cycling program: 1 cycle at 95 °C for 10 min, followed by 40 amplification cycles, each cycle consisting of a denaturation step at 95 °C for 10 s, primer annealing at 58 °C for 25 s, and extension at 72 °C for 25 s. QPCR results were normalized to the expression of the housekeeping genes GAPDH (RefSeq_ID: NM_0002046.3), ACTB (RefSeq_ID: NM_007393) and YWHAZ (RefSeq_ID: NM_003406.2). Differential expression was calculated using the ∆CP method. The differential expression was further analysed by a pairwise Wilcoxon signed rank test using the JMP®-software (JMP, A Business Unit of SAS, Cary, USA). The overall alpha level was set at 0.05.

**Table 1:** Primer information: sequences and length of the amplified fragment

Gene	RefSeq_ID	Primer sequences (sense and antisense)	Size
COL10A1	NM_000493.3	5′- CCTACTCCTTATTTACGACGCAAT-3′ 5′- TGAAAAGCCTTGAAAGAATGG-3’	107 bp
COL11A1	NM_001854.2	5’-TGATAATTTATGACAAAAGAACATACC-3′ 5’-CCAGGTAGCCAAGACTTGAGTTTA-3’	94 bp
CSPG2	NM_004385.2	5’-GAATGGGATCCTGATGGAAC-3′ 5’-AGTCCTCCATTCAGGCCTTT-3’	96 bp
GREM1	NM_013372.5	5’- TCATTTAAAAACGGCAAAGAA-3′ 5′- TTCATGAAACTTGAAGCCAAA-3’	111 bp
KRT14	NM_000526.3	5’- CAGATCCCACTGGAAGATCC-3′ 5′- AAGCTGTATTGATTGCCAGGA-3’	92 bp
MMP11	NM_005940.3	5’- AATCCAGGCCAAAAAGTTCA-3′ 5′- CCTGGGACAGGATTGAGGTA-3’	100 bp
PLEKHC1	NM_006832.1	5’-GGCCATGTTCTAGTCTGTTGC-3′ 5’-CTCTCCCTCGCACCCTTT-3’	93 bp
SULF1	NM_015170.1	5’- CAAATTAGCTGCTTGCCTGA-3′ 5′- AACTTGAAATCTTTTTACAAAGCACA-3’	99 bp
THBS2	NM_003247.2	5’-AGGTTGATGAAACGTCATGTG-3′ 5’-AAGTGCAGGGTTTCAGTGGT-3’	93 bp
GAPDH	NM_002046.3	5’-CTCCTCACAGTTGCCATGTA-3′ 5′- GCACAGGGTACTTTATTTGATGG-3’	90 bp
ACTB	NM_007393	5’-TCCCCCAACTTGAGATGTATGAAG-3′ 5’-AACTGGTCTCAAGTCAGTGTACAGG-3’	90 bp
YWHAZ	NM_003406.2	5’- TGGAGGGTCGTCTCAAGTAT-3′ 5′- GCTCCGTCTCAATTTTCTCTC-3’	94 bp

## Results

### Tissue selection and laser microdissection

Tissue samples were obtained from a total of 71 breast cancer patients. 15 tumours containing DCIS and IBC were selected from the FFPE tissue bank of the Institute of Pathology, University Hospital Tuebingen, Germany (Table 2). Five of these tissues correspond to cryopreserved specimen which has already been used in the fresh frozen investigation. For validation of differentially expressed transcripts, DCIS and IBC areas from additional 25 DCIS/IBC tumours, selected from the FFPE tissue bank of the Institute of Pathology, Ludwig Maximilians University Munich, Germany, were processed (Table 2). Tissue sections before and after the LCM procedures of a representative case are shown in Fig. 1. In order to identify potential candidate transcripts that differentiate high-risk from low-risk DCIS, the microdissected DCIS areas from 31 patients with pDCIS were used for qPCR analysis (Table 3).

**Table 2:** Histopathological data and intrinsic subtypes of DCIS/IBC tumour samples included in the test-set (upper panel) and validation-set (lower panel; T = Tuebingen; M = Munich; n.d. = not done). The risk categories are calculated based on individual ROR (risk-of-recurrence) scores taking the lymph node status into account. For N0 patients, low-risk, ROR 0–40 (10-years distant recurrence rate < 10%), intermediate-risk, ROR 41–60 (10-years distant recurrence rate 11–20%), high-risk, ROR 61–100 (10-years distant recurrence rate > 20%). For N1 patients, low-risk, ROR 0–15, intermediate-risk, ROR 16–40, high-risk, ROR 41–100, Samples with an asterisk are similar to samples from the fresh frozen set

sample #	Age	T	Grade	N	M	ER	PR	HER2	PAM50 intrinsic subtype (ROR)	Prosigna risk category
Histopathological data and intrinsic subtypes of DCIS/IBC tumours of the test-set										
T DCIS/IBC 1	52	pT1c	G2	pN0	Mx	neg	neg	neg	luminal A (0)	low
T DCIS/IBC 2	73	pT1c	G2	pN0	M0	pos	pos	neg	luminal A (25)	low
T DCIS/IBC 3	52	pT2	G2	pN0	Mx	pos	pos	neg	luminal A (32)	low
T DCIS/IBC 4	66	pT1c	G2	pN0	Mx	pos	neg	neg	luminal A (30)	low
T DCIS/IBC 5	67	pT2	G2	pN1a	M0	pos	pos	neg	luminal A (34)	low
T DCIS/IBC 6	68	pT2	G2	pN2a	Mx	pos	pos	neg	luminal A (33)	low
T DCIS/IBC 7	45	pT2	G2	pN1a	Mx	pos	pos	neg	luminal A (27)	low
T DCIS/IBC 8*	57	pT1	G2	pN0	M0	pos	pos	pos	luminal A (55)	intermediate
T DCIS/IBC 9*	72	pT1c	G3	pN0	M0	pos	pos	neg	luminal B (80)	high
T DCIS/IBC 10	45	pT1c	G2	pN0	M0	neg	neg	neg	HER2-enriched (71)	high
T DCIS/IBC 11*	74	pT1c	G2	pN2a	M0	pos	pos	neg	HER2-enriched (93)	high
T DCIS/IBC 12*	59	pT2	G2	pN2a	M0	neg	neg	neg	basal-like (64)	high
T DCIS/IBC 13	54	pT2	G3	pN0	M0	neg	neg	neg	basal-like (59)	intermediate
T DCIS/IBC 14	49	pT2	G2	pN1a	M0	neg	neg	neg	n.d.	n.d.
T DCIS/IBC 15*	66	pT2	G2–3	pN0	M0	neg	neg	pos	n.d.	n.d.
Histopathological data and intrinsic subtypes of DCIS/IBC tumours of the validation-set										
M DCIS/IBC 1	51	pT1c	G2	pN0	M0	pos	pos	neg	luminal A (0)	low
M DCIS/IBC 2	62	pT1b	G2	pN0	Mx	pos	neg	neg	luminal A (9)	low
M DCIS/IBC 3	58	pT1b	G2	pN0	Mx	pos	pos	neg	luminal A (11)	low
M DCIS/IBC 4	45	pT1c	G1	pN0	M0	pos	pos	neg	luminal A (12)	low
M DCIS/IBC 5	62	pT1b	G2	pN0	Mx	pos	pos	neg	luminal A (20)	low
M DCIS/IBC 6	67	pT1c	G2	pN0	M0	pos	pos	neg	luminal A (26)	low
M DCIS/IBC 7	45	pT1b	G2	pN0	Mx	pos	pos	neg	luminal A (27)	low
M DCIS/IBC 8	63	pT1c	G2	pN0	M0	pos	pos	neg	luminal A (30)	low
M DCIS/IBC 9	45	pT1b	G2	pN0	Mx	pos	pos	neg	luminal A (32)	low
M DCIS/IBC 10	59	pT1c	G2	pN0	M0	pos	pos	neg	luminal A (32)	low
M DCIS/IBC 11	68	pT1b	G2	pN0	Mx	pos	pos	neg	luminal A (33)	low
M DCIS/IBC 12	54	pT1a	G2	pN0	Mx	pos	pos	neg	luminal A (33)	low
M DCIS/IBC 13	61	pT1b	G2	pN0	Mx	pos	pos	neg	luminal A (33)	low
M DCIS/IBC 14	60	pT1c	G2	pN0	Mx	pos	pos	neg	luminal A (40)	low
M DCIS/IBC 15	65	pT1c	G3	pN0	M0	pos	pos	neg	luminal A (43)	intermediate
M DCIS/IBC 16	40	pT1c	G2	pN1a	M0	pos	pos	neg	luminal A (30)	low
M DCIS/IBC 17	65	pT1c	G2	pN0	M0	pos	pos	neg	luminal A (53)	intermediate
M DCIS/IBC 18	58	pT1c	G3	pN0	Mx	pos	pos	pos	luminal A (76)	high
M DCIS/IBC 19	67	pT1c	G2	pN2a	M0	pos	pos	neg	luminal A (36)	low
M DCIS/IBC 20	42	pT1b	G3	pN1a	Mx	pos	pos	neg	luminal A (48)	intermediate
M DCIS/IBC 21	57	pT1c	G3	pN0	M0	pos	pos	neg	luminal A/B (31/58)	intermediate
M DCIS/IBC 22	51	pT1c	G3	pN0	M0	pos	pos	neg	HER2-enriched (77)	high
M DCIS/IBC 23	62	pT1c	G3	pN1mi	M0	pos	pos	pos	HER2-enriched (77)	high
M DCIS/IBC 24	61	pT1c	G2	pN0	M0	pos	pos	neg	n.d.	n.d.
M DCIS/IBC 25	28	pT1b	G3	pN1a	Mx	neg	pos	pos	n.d.	n.d.

**Table 3:** Histopathological data and intrinsic subtypes of pure DCIS tumour samples (n.d. = not done)

Histopathological data and intrinsic subtypes of pure DCIS									
sample #	Age	T	Grade	N	M	ER	PR	HER2	10-year recurrence-free survival
pDCIS 1	49	pTis	G3	pN0	M0	pos	pos	neg	low
pDCIS 2	61	pTis	G3	pN0	M0	pos	pos	neg	low
pDCIS 3	54	pTis	G3	pN0	M0	pos	pos	neg	low
pDCIS 4	44	pTis	–	pN0	M0	pos	pos	neg	low
pDICS 5	32	pTis	G3	pN0	M0	pos	pos	neg	low
pDCIS 6	65	pTis	G3	pN0	M0	neg	neg	neg	low
pDCIS 7	63	pTis	G3	pN0	M0	pos	pos	neg	low
pDCIS 8	36	pTis	G3	pN0	M0	pos	pos	neg	low
pDCIS 9	36	pTis	G3	pN0	M0	pos	pos	neg	low
pDCIS 10	63	pTis	G3	pN0	M0	pos	pos	neg	low
pDCIS 11	43	pTis	G3	pN0	M0	pos	pos	neg	low
pDCIS 12	46	pTis	G3	pN0	M0	pos	pos	neg	low
pDCIS 13	84	pTis	G2	pN0	M0	pos	pos	neg	low
pDCIS 14	55	pTis	G2	pN0	M0	pos	pos	neg	low
pDCIS 15	46	pTis	G2	pN0	M0	pos	pos	neg	low
pDCIS 16	79	pTis	G3	pN0	M0	neg	neg	pos	low
pDCIS 17	46	pT1	G3	pN0	M0	pos	pos	neg	intermediate
pDCIS 18	84	pTis	G2	pN0	M0	pos	pos	neg	low
pDCIS 19	53	pTis	G3	pN0	M0	pos	pos	neg	intermediate
pDCIS 20	46	pTis	G3	pN0	M0	neg	pos	pos	intermediate
pDCIS 21	33	pTis	G2	pN0	M0	pos	pos	neg	intermediate
pDCIS 22	71	pTis	G3	pN0	M0	pos	neg	neg	high
pDCIS 23	65	pTis	G3	pN0	M0	pos	neg	neg	high
pDCIS 24	59	pTis	G3	pN0	M0	neg	pos	pos	intermediate
pDCIS 25	65	pTis	G3	pN0	M0	neg	neg	pos	intermediate
pDCIS 26	47	pTis	G3	pN0	M0	neg	neg	pos	low
pDCIS 27	46	pTis	G3	pN0	M0	pos	neg	pos	intermediate
pDCIS 28	53	pTis	G3	pN0	M0	pos	pos	pos	intermediate
pDCIS 29	53	pT1b	G3	pN0	M0	neg	neg	neg	low
pDCIS 30	40	pTis	G3	pN0	M0	neg	neg	pos	n.d.
pDCIS 31	49	pTis	G3	pN0	M0	neg	neg	neg	n.d.

The Prosigna™ Breast Cancer Prognostic Gene Signature Assay was performed for 13 out of 15 and 23 out of 25 FFPE samples and classified the majority of the IBC tumour samples of our study as luminal A subtype and determined a low risk of recurrence for them (Tables 2 and 3).

### Selection of candidate genes

**Fig. 2:**
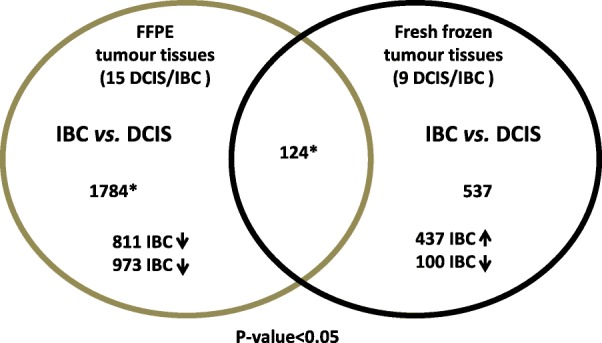
Comparison of FFPE and fresh frozen gene sets. The Venn-diagram illustrates the overlap of differentially expressed transcripts in both FFPE experiment (*n* = 15) and the fresh frozen (*n* = 9) experiment [18]. Genes marked with an asterisk are listed in Additional files 1 and 2. Arrows indicate up- or downregulated in IBC compared to the patient matched DCIS component

WG-DASL analysis in the 15 FFPE test set DCIS/IBC samples revealed 1784 transcripts that were differentially expressed between DCIS and IBC (*P* < 0.05; Additional file 1). Comparison of them with the differentially expressed transcripts from the fresh frozen investigation left 124 transcripts with analogous expression in both investigations (*P* < 0.05; Fig. 2; Additional file 2). In a previously performed network analysis using the Ingenuity® software and the significant differentially expressed transcripts obtained in the fresh frozen investigation we selected 9 genes that are involved in cellular processes such as cell-to-cell adhesion, extracellular matrix organization, metastasis, and tumour progression (Table 4): THBS2 (thrombospondin 2; RefSeq_ID: NM_003247.2), GREM1 (gremlin 1; RefSeq_ID: NM_013372.5), MMP11 (matrix-metalloproteinase 11; RefSeq_ID: NM_005940.3), COL11A1 (collagen type XI-alpha 1; RefSeq_ID: NM_001854.2), COL10A1 (collagen type X-alpha 1; RefSeq_ID: NM_000493.3), CSPG2 (chondroitin sulphate proteoglycan 2; RefSeq_ID: NM_004385.2), PLEKHC1 (pleckstrin homology domain containing member 1; RefSeq_ID: NM_006832.1), SULF1 (sulfatase 1; RefSeq_ID: NM_015170.1) and KRT14 (cytokeratin 14; RefSeq_ID: NM_000526.3). The expression of these transcripts was further investigated. In the 15 FFPE test set DCIS/IBC samples the same significant expression difference (upregulation) was obtained for COL11A1, MMP11, THBS2, CSPG2, and GREM1. Also the significant downregulation (*P* < 0.05) of KRT14 in IBC compared to DCIS could be verified. For COL10A1 (*P* < 0.06) and SULF1 (0.053) strong tendencies to upregulation in IBC were achieved. Only the differential expression of PLEKHC1 did not reach statistical significance (*P* > 0.5) in the FFPE investigation (Table 4). However, we decided to further include all 9 transcripts in the following validation experiments.

**Table 4:** Selected candidate genes for validation using qPCR. The progression-associated genes were identified after analysis of the fresh frozen investigation and are significantly differentially expressed between DCIS and IBC of the same tumours. Six of them are also differentially expressed between DCIS and IBC of the investigated FFPE tumours. The differential expressions of COL10A1 of SULF1 are almost significant. Differential expression of PLEKHC1 could not be verified

		Fresh frozen investigation		FFPE investigation	
Gene	RefSeq_ID	logFC	*p*-value	fold change	t-test *p*-value
COL10A1	NM_000493.3	5.11	4.34E-05	1.86	0.06
MMP11	NM_005940.3	5.09	4.92E-06	2.31	0.0042
COL11A1	NM_001854.2	3.64	4.75E-05	1.96	0.0006
THBS2	NM_003247.2	3.50	4.34E-05	1.02	0.0479
CSPG2	NM_004385.2	3.23	0.00126	2.74	0.0033
GREM1	NM_013372.5	3.02	7.36E-05	1.85	0.0033
SULF1	NM_015170.1	2.15	0.000101	1.40	0.0553
PLEKHC1	NM_006832.1	1.84	0.000101	0.33	0.5244
KRT14	NM_000526.3	−4.00	0.00433	−1.74	0.0015

### Verification of microarray data by qPCR in IBC tumour samples

**Fig. 3:**
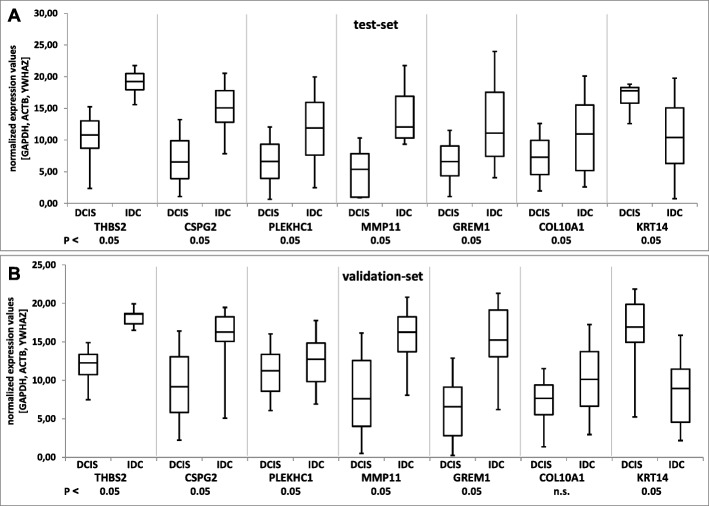
Validation of differential expression of transcripts in DCIS/IBC-tumours by qPCR. **a**) Test-set: The selected progression-associated genes show a significant difference in expression between DCIS and IBC of the same tumour (*P* < 0.05). **b**) Validation-set: Except for COL10A1, all genes show significant differential expression, confirming the results of the technical validation set (*P* < 0.05). PCR values are normalized to GAPDH, ACTB and YWHAZ

To validate the WG-DASL findings, qPCR-based relative quantification was performed for the 9 selected transcripts using first the same 15 LCM-isolated tissue samples that have been selected for WG-DASL profiling (test-set). In agreement with the findings of WG-DASL gene expression profiling, qPCR confirmed significant upregulation of THBS2, CSPG2, MMP11, GREM1 and COL10A1 and downregulation of KRT14 in IBC compared to the corresponding DCIS (Fig. 3a). While the difference in PLEKHC1 expression was not significant in the WG-DASL profiling experiment using the same FFPE tissues qPCR showed PLEKHC1 expression to be significantly upregulated in IBC (Fig. 3a).

Similar findings were obtained using further 25 laser microdissected DCIS and IBC pairs (validation-set). Statistically significant expression differences were found for THBS2, CSPG2, PLEKHC1, MMP11, GREM1 and KRT14 (*P* < 0.05) whereas for COL10A1 this time only a trend to increased expression in IBC was observed (Fig. 3b).

### Identification of high–risk DCIS

**Fig. 4:**
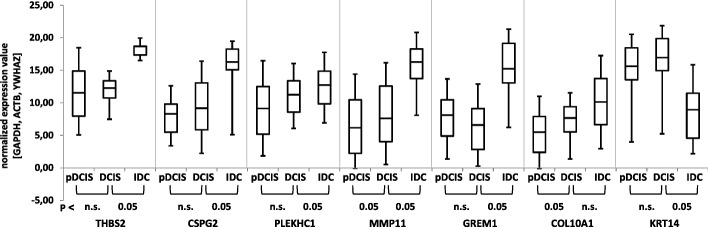
Independent validation of differential expression of the gene set in pDCIS and DCIS/IBC-tumours by qPCR. Expression of MMP11 increases significantly from pDCIS to DCIS of DCIS/IBC mixed tumours and IBC (*P <* 0.05) and COL10A1 increases significantly from pDCIS to DCIS of DCIS/IBC mixed tumours (*P <* 0.05). Differences in expression of the remaining genes are not significant (n.s.). PCR values are normalized to GAPDH, ACTB and YWHAZ

We hypothesise that genes exhibiting a continuous increase or decrease in their expression at least from pDCIS to DCIS (of DCIS/IBC mixed tumours) and ideally further to IBC may be able to discriminate high-risk DCIS from low-risk DCIS. Therefore, the expression of the selected candidate genes was also determined in 31 laser microdissected pDCIS tissue samples. All selected target transcripts could be detected in pDCIS. For THBS2, CSPG2, GREM1 and KRT14, expression differences between pDCIS and DCIS were either not significant or did not follow a continuous trend of up- or downregulation from pDCIS to DCIS. Only the expression of COL10A1 and MMP11 was found to progress from ‘low expression’ in pDCIS, to ‘intermediate expression’ in DCIS and ‘high expression’ in the corresponding IBC, the difference between pDCIS and DCIS (of DCIS/IBC mixed tumours) being significant (*P* < 0.05; Fig. 4).

## Discussion

In patients with DCIS the risk of progression to IBC is the most important consideration in selecting the appropriate management of these lesions [32]. A decisive event in breast cancer progression is the transition of tumour cells through the basement membrane into the surrounding stromal compartment. The underlying molecular events are poorly understood. Identification of genes and proteins driving this process could serve as prognostic markers and therapeutic targets. In order to address the question whether identification of molecular biomarkers for high-risk DCIS is possible we have determined transcripts, which are differentially expressed between the DCIS and IBC component found in DCIS/IBC-tumours and investigated whether such candidate transcripts are even differently represented in pDCIS that did not progress to IBC within 5 to 10 years of follow-up.

One challenge to this is, however, that for reasons of diagnostic certainty, pDCIS specimens are only rarely cryopreserved. Instead, pDCIS samples from FFPE tissue banks are widely available, but are of poorer RNA quality. Technical progress has been achieved to enable gene expression profiling of archival FFPE samples, but it still remains extremely challenging. We have applied gene expression profiling using WG-DASL Assay and qPCR to analyse epithelial cells of pDCIS, DCIS and their corresponding IBC in FFPE breast cancer tissue in the context of tumour progression. The findings were compared with results from a similar study and in which we have investigated cryopreserved breast cancer tissue containing DCIS and IBC areas [18] with the aim to independently cross-validate both expression data.

WG-DASL analysis in the 15 FFPE test set DCIS/IBC samples revealed 1784 transcripts that are differentially expressed between DCIS and IBC of which 124 showed concordant expression in cryopreserved tissues in which 537 total transcripts had been found to be differentially expressed between DCIS and IBC. The resulting overlap of 23% is quite surprising since it is known that gene expression data generated with different array platforms generally show only a marginally overlap [33]. It suggests that our approach can provide concordant data and that the two data sets can be used for reciprocal confirmation. Further assuring is the fact that among the overlapping genes are transcripts such as MEF2C, LRRC15, BGN, BPAG1, OLFML2B, POSTN, THBS2, PLEKHC1, COL11A1 and FAP, which have already been associated with tumour progression/invasion [18, 34–38].

In order to identify a minimal gene set that may be applicable for routine clinical use by e.g. PCR to distinguish between DCIS and IBC the fresh frozen data set from Schuetz et al. [18] could be concentrated by statistical means resulting in nine transcripts. Eight of them are upregulated in IBC (THBS2, CSPG2, PLEKHC1, GREM1, MMP11, COL10A1, COL11A1, SULF1) and one is downregulated (KRT14) (Table 4; Additional file 3). Of these analogous and significant differential expression was verified for six transcripts by the DASL assay in all 15 DCIS/IBC pairs in the FFPE investigation. For two other transcripts the concordances were almost significant and only the differential expression of PLEKHC1 could not be verified (Table 4).

When validating the expression levels of the nine selected transcripts using qPCR in both the microdissected tissue specimen used for DASL gene expression profiling (test-set, *n* = 15) and in an independent set of further 25 laser microdissected DCIS/IBC pairs (validation-set) seven of them could be confirmed. Only two transcripts, SULF1 and COL11A1, did not produce conclusive PCR results. It is difficult to estimate with confidence whether this validation rate is adequate to draw conclusions, given the relatively limited number of published WG-DASL studies on FFPE tissues and the FFPE-nature and therefore highly variable quality of the starting material. We would recommend validation using a larger sample cohort. However, in summary, our results indicate that differential expression of most transcripts belonging to the minimal progression-associated gene set can be verified in an independent sample cohort.

Some of our candidate transcripts are involved in biological processes related to extracellular matrix remodelling [39]. However, these candidate genes, especially MMP11 and COL11A1, were all detected in microdissected DCIS (of DCIS/IBC mixed tumors) and in pDCIS where we could safely and completely isolate tumor cells from the surrounding stroma, therefore proving the expression of these genes in neoplastic epithelial cells. In IBC tissues, where the epithelial complexes are much smaller, we estimated to enrich the tumor cell compartment to a purity of at least 80% using LCM. Therefore we do not think that the upregulated expression we detected for these candidate genes could only be caused by ´contamination` of co-microdissected stromal cells. Immunohistochemical investigations for the in situ detection of candidate gene on the protein level would have increased the value of our findings, but were beyond the scope of the present study.

One explanation for the detection of genes which are regarded to be of stromal or fibroblast origin might be that tumour progression is thought to include processes such as epithelial-mesenchymal transition (EMT), whereby epithelial cells lose epithelial polarity, acquire a fibroblastoid phenotype and loose cell-to-cell-adhesion [40]. In support of this some of the putatively stroma-derived transcripts can be found in e.g. ‘core EMT interactome gene-expression signature’ in which the transcript for KRT14 is downregulated and transcripts for GREM1, PLEKHC1 and several collagens and MMPs are upregulated [41]. Also the ‘EMT core signature’ defined by Anastassiou et al. [41] contains GREM1 and several other genes - e.g. DCN, SPARC, INHBA, MMP13, and PDGFRB – which are represented in our FFPE data set. The role of EMT in breast cancer prognosis is still under debate; however, a number of EMT-related genes have been linked to poor outcome in breast cancer [42, 43].

In support of our data some of the differentially expressed genes represented in our progression-associated candidate set (COL10A1, COL11A1, MMP11, SULF1, and THBS2) have also been identified in another study comparing gene expression of matched DCIS/IBC pairs [14]. During breast cancer progression MMP11 expression is significantly increased in IBC compared to DCIS, supporting our data that it may be a key player driving the DCIS-to-IBC transition [39]. Vargas et al. [44] also observed genes such as COL11A1, COL5A2 and MMP13 in epithelial cells of IBC compared to DCIS.

MMP11 - also called stromelysin 3 – has already been associated with the invasion of tumour cells and is a marker of poor prognosis [18, 45, 46]. The **COL10A1** gene encodes the alpha chain of type X collagen, a short chain collagen expressed by hypertrophic chondrocytes during endochondral ossification. Its expression is greatly increased in breast cancer tissue compared to normal breast tissue [47].

Of at least equal value for the clinical management of breast cancer would be information on the prognosis with regard to tumour progression at the DCIS stage. Therefore, one important aspect of this work is that pDCIS samples were used to verify if some of the 9 selected transcripts follow a continuous trend of up- or downregulation from pDCS to DCIS and may thus be able to discriminate between high-risk and low-risk DCIS. Besides expected inter-patient expression differences due to different origin of pDCIS and DCIS with IBC component, MMP11 and COL10A1, significantly progressed from ‘low expression’ in pDCIS, to ‘intermediate expression’ in DCIS and further to ‘high expression’ in the corresponding IBC, with the differences between pDCIS and DCIS of DCIS/IBC mixed tumours. The fact that the differences in COL10A1 expression did not reach significance in the independent validation cohort might be caused by the low sample numbers.

## Conclusion

By validation of microarray gene expression data using LCM samples from cryopreserved and FFPE DCIS/IBC breast cancer tissues, we identified candidate progression-associated transcripts which might be important for the transition of breast epithelial cells from DCIS to IBC. In addition, the inclusion of pDCIS tissues revealed MMP11 and COL10A1 as potential indicators of high-risk DCIS.

## Additional files

Additional file 1: WG-DASL analysis resulted in 1784 transcripts that were differentially expressed between DCIS and IBC in the FFPE breast cancer tissue samples (*P* < 0.05). (XLS 193 kb)

Additional file 2: Overlapping genes, Description: Comparison of WG-DASL results with the data from the FF investigation. 124 of the transcripts were found to show differential expression in both investigations (*P* < 0.05). (XLS 41 kb)

Additional file 3: Validation 5 FFPE samples analogue to FF samples, Description: A) Analogue samples FFPE-Cryo: The selected progression-associated genes are significantly differential expressed between DCIS and IBC of the same tumour (*P* < 0.05; n.s. = not significant). B) Remaining FFPE samples: Except for COL10A1, all genes are significantly differential expressed and confirm the results of the technical validation set (*P* < 0.05; n.s. = not significant). PCR values are normalized to GAPDH, ACTB and YWHAZ (PDF 45 kb)

## Abbreviations

DASL: cDNA-mediated annealing, selection, extension and ligation
DCIS: Ductal carcinoma in situ
FF: Fresh frozen
FFPE: Formalin-fixed paraffin-embedded
IBC: Invasive duct carcinoma
LCM: Laser capture microdissection
pDCIS: Pure DCIS
