# Supplementary material for: Progression-specific genes identified in microdissected formalin-fixed and paraffin-embedded tissue containing matched ductal carcinoma in situ and invasive ductal breast cancers
Source: BMC Med Genomics. 2018 Sep 20;11:80. doi: 10.1186/s12920-018-0403-5 (PMC6147035; doi:10.1186/s12920-018-0403-5)
Supplement: Supplementary file 3 — Validation 5 FFPE samples analogue to FF samples, Description: A) Analogue samples FFPE-Cryo: The selected progression-associated genes are significantly differential expressed between DCIS and IBC of the same tumour (P < 0.05; n.s. = not significant). B) Remaining FFPE samples: Except for COL10A1, all genes are significantly differential expressed and confirm the results of the technical validation set (P < 0.05; n.s. = not significant). PCR values are normalized to GAPDH, ACTB and YWHAZ (PDF 45 kb) [file 12920_2018_403_MOESM3_ESM.pdf]

Additional file 3

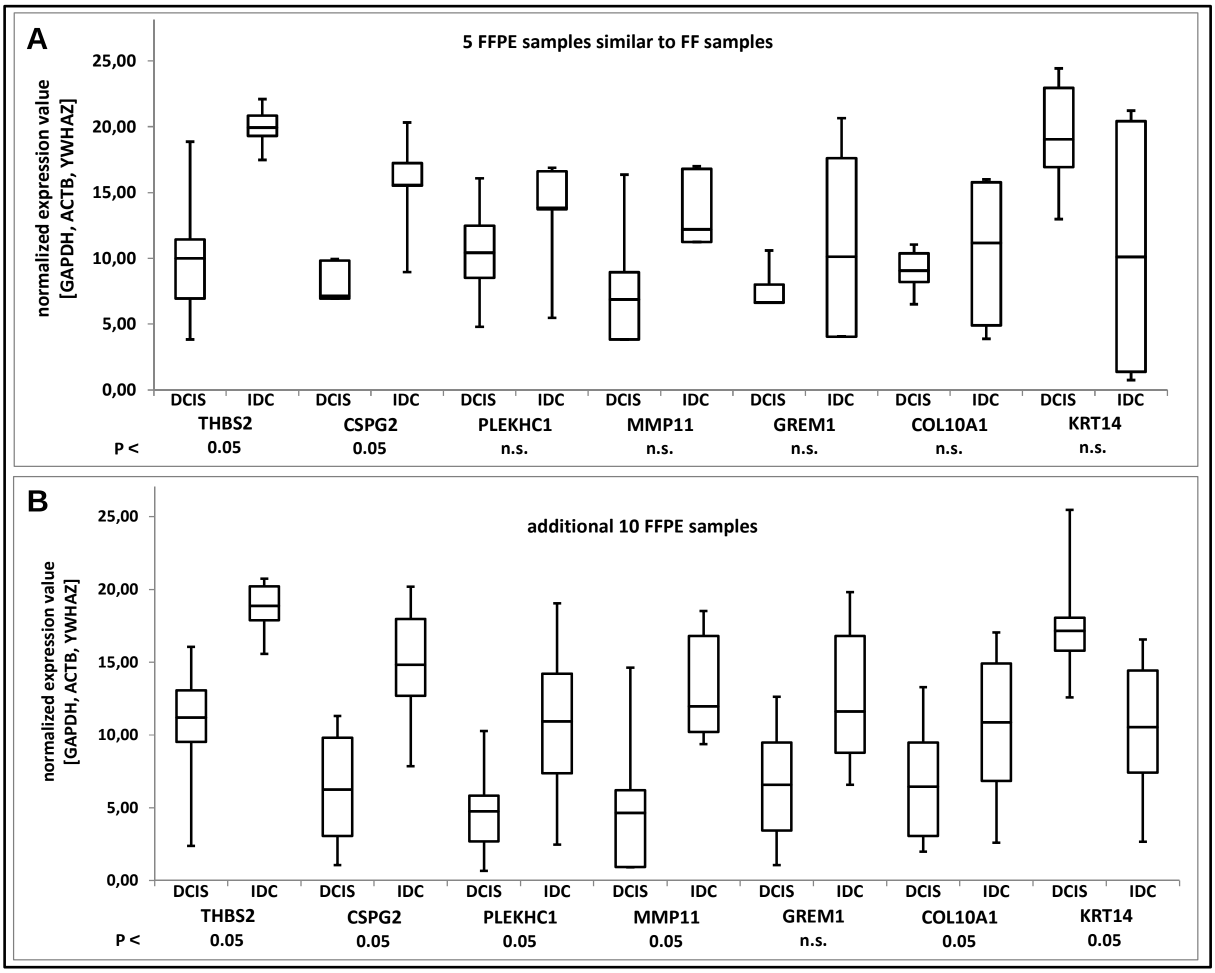

Validation 5 FFPE samples analogue to FF samples. A) Analogue samples FFPE-Cryo: The selected progression-associated genes are significantly differential expressed between DCIS and IDC of the same tumour ($P<0.05$; n.s. = not significant). B) Remaining FFPE samples: Except for COL10A1, all genes are significantly differential expressed and confirm the results of the technical validation set ($P<0.05$; n.s. = not significant). PCR values are normalized to GAPDH, ACTB and YWHAZ
